# Treatment of knee cartilage lesions in 2024: From hyaluronic acid to regenerative medicine

**DOI:** 10.1002/jeo2.12016

**Published:** 2024-04-02

**Authors:** Mats Brittberg

**Affiliations:** ^1^ Cartilage Research Unit, Team Orthopedic Research Region Halland‐TOR, Region Halland Orthopaedics, Varberg Hospital University of Gothenburg Varberg Sweden

**Keywords:** cartilage repair, chondrogeneic cells, direct repair, indirect repair, orthobiologics

## Abstract

**Abstract:**

Intact articular cartilage plays a vital role in joint homeostasis. Local cartilage repairs, where defects in the cartilage matrix are filled in and sealed to congruity, are therefore important treatments to restore a joint equilibrium. The base for all cartilage repairs is the cells; either chondrocytes or chondrogeneic cells from bone, synovia and fat tissue. The surgical options include bone marrow stimulation techniques alone or augmented with scaffolds, chondrogeneic cell implantations and osteochondral auto‐ or allografts. The current trend is to choose one‐stage procedures being easier to use from a regulatory point of view. This narrative review provides an overview of the current nonoperative and surgical options available for the repair of various cartilage lesions.

**Level of Evidence:**

Level IV.

**Abbreviations**
ACI: autologous chondrocyte implantation
AMIC: autologous matrix‐induced chondrogenesis
BMAC: bone marrow aspirate concentrates
BMS: bone marrow stimulation
CAFRIMA: cartilage fragment implantation membrane augmented
ECM: extracellular matrix
HA: hyaluronic acid
MOCART: Magnetic Resonance Observation of Cartilage Repair Tissue system
MRI: magnetic resonance imaging
MSCs: mesenchymal stem cells
OA: osteoarthritis
PRP: platelet‐rich plasma
RCTs: randomized clinical trials

## INTRODUCTION

Joint homeostasis involves the balance of various factors to ensure the optimal function and health of the joint structures [39, 88]. When a joint is injured, it is important to restore the disturbed equilibrium. Injuries in the knee joint include the cartilage, subchondral bone, the menisci, ligaments and tendons [6]. Very seldom only one of those structures is damaged. Hjelle et al. [45] looked on 1000 arthroscopies and found that local chondral or osteochondral defects were found in 19% of the patients. In those patients, 61% related their current knee problem to a previous trauma, and a concomitant meniscal or anterior cruciate ligament injury was found in 42% and 26%, respectively [45]. To restore a disturbed homeostasis, all those injured structures' damage may then need to be addressed.

Injuries limited to only the matrix have the potential for restoration of the matrix by chondrocyte matrix synthesis [38, 66]. If such injuries also involve chondrocyte death, spontaneous repair is limited and results in a matrix with a changed structure [59]. Furthermore, if the chondrocytes are not able to synthesize new matrix, the damaged matrix loses proteoglycans, resulting in cartilage with decreased ability to resist mechanical forces [74].

The treatment of the damaged cartilage could be divided into indirect or direct repairs. When different injections are used to stimulate repair mechanisms and reduce inflammation, an indirect repair effect may be seen facilitating local cartilage repair. A direct repair is a direct treatment in the lesion site.

To be successful when treating patients with cartilage lesions, one should give the patients a high percentage of symptom relief with pain reduction and functional recovery. Another goal is to hinder or slow down a potential progression into osteoarthritis (OA).

Cartilage repair today involves filling up and sealing off a defect area of the joint surface, being either a chondral or osteochondral repair.

The filling should be:

- Resistant to wear.
- Reduce loading forces on the subchondral bone.

Furthermore, a one and only cartilage repair technique does not exist. Subsequently, the surgeon's choice of treatment should be based on several variables, and a summary of those variables will result in the most suitable treatment for the patient.

**Figure 1:**
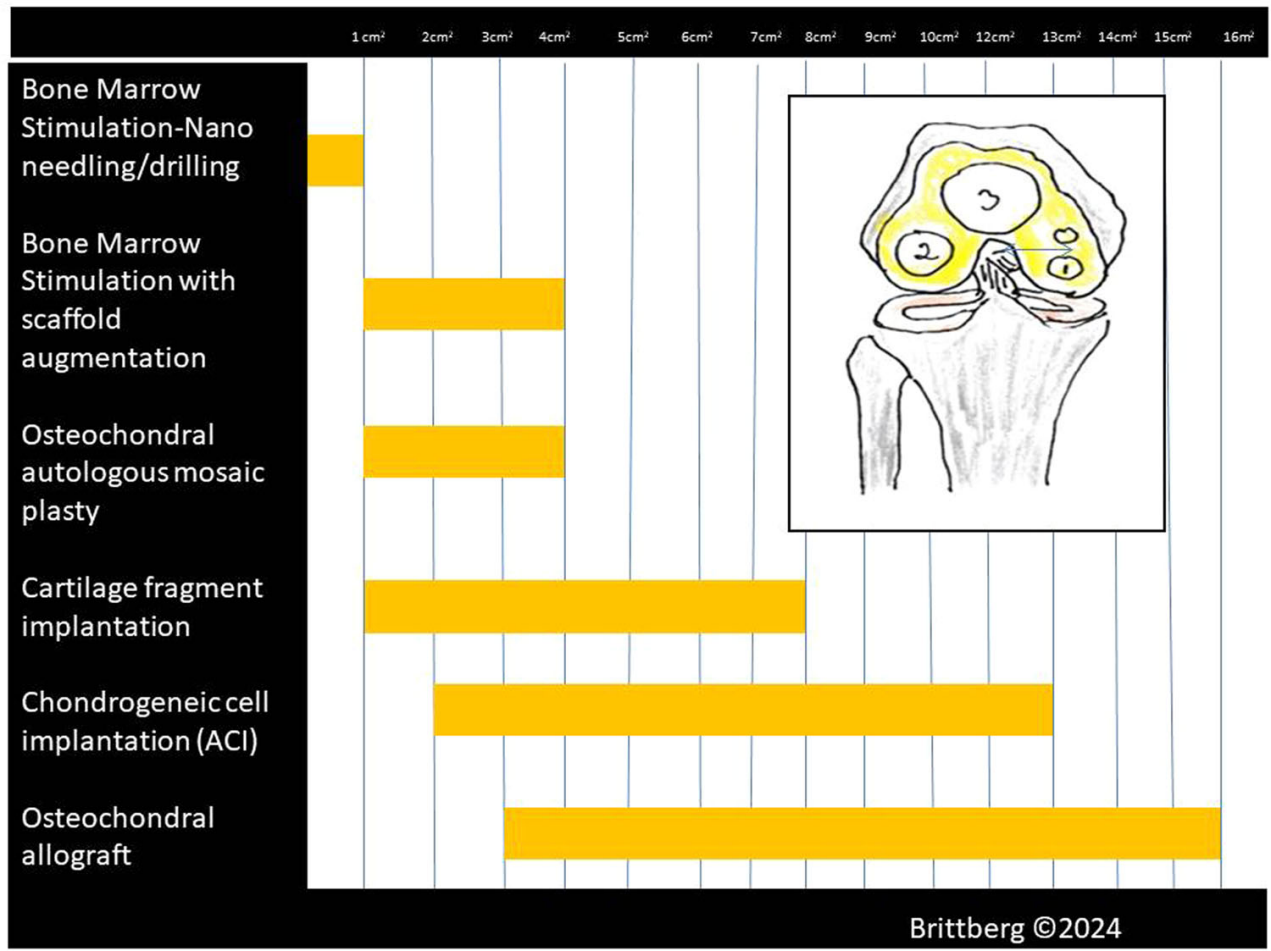
Proposed treatment algorithm related to cross‐section areas. Remember that in a recent study on MRI, the mean width of the medial condyle has been found to have a mean of 26.2 mm, and on the lateral condyle, the mean widths were 32.5 mm [103]. A defect with a cross‐sectional area of 1 cm^2^ on the central part of one of the condyle is then a rather large defect. When treating cartilage defects, it is then important to understand the treatment choice's ability to fill up a defect with a repair tissue and by that unload the surroundings as well.

Important variables are:

*Lesion size*: When it comes to the choice of surgical method for a specific cartilage injury, it is the size of the injury that is most important for governing the repair selection [103]. For example, when treating a medial femur condyle injury, when you know the width of the condyle, it is easier to realize how large an injury is in relation to the loaded articular surface [103]. Improved lesion sizing is mandatory, and without a more accurate measurement of the size of the injury, the surgeon tends to overestimate the size of the injury [86, 87, 109]. See proposed treatment algorithm related to square areas in Figure 1.

*Lesion depth*: Superficial chondral lesions (less than 50% of cartilage depth) are suitable for debridement [14]. Lesions with a depth of more than 50% of the cartilage tissue thickness are suitable for either repair or restoration techniques depending on the size of lesions [14].

*Lesion surrounding cartilage quality*: The ideal cartilage lesion should have contained walls to support a repair tissue ingrowth and maturation [50, 104]. If surrounding cartilage is too thin, an unloading osteotomy might be considered in conjunction with a local repair.

*Alignment*: To maintain the homeostasis of the joint, even load on the cartilage is important. Too much load, as can be seen in varus or valgus knees, can disturb the homeostasis with cartilage breakdown [73]. Cartilage repair of lesions in a malaligned joint subsequently needs to be combined with unloading osteotomies. The main principle of correction osteotomies is then to achieve a transfer of loading from diseased areas of the joint to areas with relatively intact, healthy cartilage [58].

*Concomitant injuries*: Most often there are not only cartilage injuries to treat but often also ligament and meniscal injuries. Lack of meniscus increases the load on the cartilage area, and an unstable joint has negative influences on the cartilage repair area [10, 71].

*Obesity*: Obesity is a risk factor for both the initiation and progression of OA in weight‐bearing and nonweight‐bearing joints [76]. In such a joint, there is risk of chronic inflammation, as well as abnormal mechanical loading due to increased body weight with negative influences also on the local repair and surrounding cartilage [62, 72, 100].

*Genetics*: Genetic diseases may increase the risk of joint disorders and cartilage abnormalities. The lesion area could then be more difficult to repair due to poor collagen production and bony involvement [101].

*Gender*: Women have a higher likelihood of developing OA compared to men. There are clear sex‐based variances in cartilage degeneration and regeneration, but the underlying mechanisms and exact effects still need further exploration [81].

*Smoking*: Literature suggests an overall negative influence on cartilage repair and highlights the need for further investigations [18, 57].

*Concomitant disease*: The presence of other diseases can affect repair. It could be diabetes, cardiovascular diseases and joint‐related immune diseases affecting the joint metabolism and surrounding bone and muscle functions [27].

The aim of this review is to give an update of what cartilage repair treatment alternatives are available in 2024 and how to use them related to certain variables.

## TREATMENT CHOICES

### Indirect repairs

Intra‐articular growth factor stimulation may be used to stimulate repair from surrounding cartilage, synovia and bone without any additional surgical intervention.

Hyaluronic acid (HA) in different gel forms may interact with cell surface receptors, support the growth of chondrocytes and promote the differentiation of mesenchymal stem cells (MSCs) to chondrocytes [1]. The effectiveness of intra‐articular therapies, such as HA gels, is limited by their fast clearance [105]. There is a need for safe formulations which could provide extended and sustained drug availability. HA injections are mainly for OA joints, but HA gels mixed with stem cells as an adjunct to arthroscopic bone marrow stimulation (BMS) for knee cartilage defects have been tested in a comparative study [95]. Complete repair with cartilage filling was achieved in 36.8% of the knees in bone marrow‐stimulated HA‐augmented group, whereas only 16.6% of the knees in group treated by BMS showed complete filling according to MOCART (Magnetic Resonance Observation of Cartilage Repair Tissue system) at 24 months [95].

Growth factors are different biologically active polypeptides that can stimulate cellular division, growth and differentiation [36]. In articular cartilage, a large number of growth factors cooperate to regulate the homeostasis of articular cartilage [36]. Most studied and tested for clinical use are transforming growth factor‐β1, bone morphogenetic protein‐2, bone morphogenetic protein‐7, IGF‐1, fibroblast growth factor‐2, fibroblast growth factor‐18 and platelet‐derived growth factor [26, 91]. Multiple growth factor/cytokine modulation therapies are currently undergoing clinical trial investigations but then only for OA.

Platelet‐rich plasma (PRP) could deliver a variety of chemical mediators, which could interact with the cells in the joint. PRP has also been used as an adjunct to local cartilage repairs [68]. There are no studies on PRP and effects on local cartilage repair in the knee. However, studies have been done to look upon the effect of PRP on cartilage volume and thickness in osteoarthritic knees [85]. Prodromidis et al. [85] looked at 14 studies with PRP injections as OA treatment. Their study included seven randomized clinical trials (RCTs) (*n* = 688), one prospective (*n* = 50), one retrospective (*n* = 68) and four case series (*n* = 224) [85]. The PRP preparation process and treatment protocol varied widely (follow‐up 6–12 months). In this meta‐analysis, PRP treatment was not associated with a significant increase in cartilage thickness (four studies, *n* = 187) [85] Meta‐analysis of three RCTs (*n* = 112) showed no significant difference in the change of overall knee cartilage content with PRP injections compared with no PRP [85]. Their conclusion was that the current literature does not support PRP as chondrogeneic treatment alternative of knee OA [85]. The limits of the conclusion were the substantial heterogeneity in the evaluated studies, which limits the power of any conclusion [85].

Stem cells and progenitor cells have a unique ability to differentiate into various cell types [19]. Progenitor cells are more committed than stem cells and have a more limited differentiation potential and can after trauma be activated to promote tissue repair and regeneration. When stem cells are used for joint injections in osteoarthritic joints, the cells act as medicinal signalling cells to counteract the ongoing chronic inflammation [19, 108].

Stem cells/progenitor cells have been used also as injections to improve local repair. One technique uses magnetic targeting for the accumulation of locally injected cells in a lesion [56]. Autologous bone marrow MSCs are then cultured and subsequently magnetized. After injection, cells could be attracted by a surrounding magnet to fill up local cartilage defects [56]. It is also possible to use injections of peripheral blood stem cells in a HA gel [89]. A recent study showed an improved outcome compared to HA and physiotherapy for massive knee chondral defects [89].

### Direct repairs

To biologically repair cartilage lesions, chondrogeneic cells are needed to induce a repair tissue. The cell sources may then be pure intrinsic by BMS or osteochondral autografts, or extrinsic as from externally manipulated cultured chondrocytes, minced cartilage and osteochondral allografts.

#### Repair by BMS with or without scaffolds

- *BMS techniques without scaffold*:

Those techniques are mainly based on different ways to induce a blood clot formation in the cartilage‐damaged area. The most common way of trying to create a healing tissue in the case of cartilage damage is to perforate the underlying bone plate in various ways and bring about bleeding and blood clot formation and induce an ingrowth of potentially cartilage‐forming ‘progenitor cells’ from the bone marrow. Through the initial Pridie technique [84] with subchondral drilling via a short period of abrasion arthroplasty [55] and a dominant long period of microfracture (MFX) technique [99], the era of deep drilling has started. Chen et al. [21, 22] have shown that MFX with an awl‐induced fracturing and bone compaction around holes that were largely sealed off from adjacent bone marrow, in contrast to drilling that cleanly removed bone debris and left open channels that communicated between the hole and marrow. Furthermore, deep drilling induced a larger subchondral haematoma compared to shallow drilling and MFX technique [21, 22]. The repair filing became better when larger vessels were reached via the subchondral bone. However, still there is a general lack of basic science literature comparing MFX versus drilling for focal chondral defects.

Experience has shown that it can be difficult to obtain a complete and an even filling of a cartilage injury after the various BMS methods described [48, 61].

- *BMS techniques with scaffold*:

In recent years, there has also been more and more interest in the MSCs in the subchondral bone. By implanting various porous materials in the debrided cartilage lesion, a stronger and more even ingrowth of cartilage‐forming cells in the cartilage damage area can be induced. Scaffolds are designed to be chondroconductive or osteoconductive. They are implanted as cell‐free constructs, most often as a three‐dimensional (3D) construct into chondral and osteochondral defects or by themselves in liquid form to augment marrow stimulation techniques.

Scaffold alternatives that are in use are:

*Collagen‐based scaffolds*: A 3D type I collagen matrix purified from rat‐tail collagen (CaReS‐1S®; Arthrokinetics) could be used in single‐stage surgery in combination with BMS [31]. In short to medium follow‐up time after surgery on small lesions, result reports have been good, while in a recent study with this technique, a failure rate of 18% after 5 years was reported in a study where the lesion size was large (a mean defect size of 3.7 ± 1.9 cm^2^) [90].

ChondroGide® (Geistlich Pharma AG) [5] is a bilayer collagen type I/III membrane [5] used in combination with BMS. Such a combination has as a technique been named autologous matrix‐induced chondrogenesis (AMIC) [5]. AMIC [5] was developed to protect the after a BMS developed blood clot and the ingrowing cells. In a study, the AMIC procedure was associated with significant improvements at 2.5 years in patients treated for knee osteochondral defects measuring 2–8 cm [82]. Furthermore, the AMIC procedure achieved greater IKDC and Lysholm score and a significant reduction of the visual analogue scale score in the management of patellar chondral defects [82]. In a randomized study, patients were randomized and treated either with MFX or with sutured or glued AMIC in a prospective multicenter clinical trial [51]. Improvement for the first 2 years was seen in all groups [107]. However, a significant score degradation was observed in the MFX group, while all scores remained stable up to 5 years in the AMIC groups. At both 2 and 5 years, MRI defect filling was more complete in the AMIC groups [107].

*HA‐based matrices*: HA‐based matrices [102] are also used to support the ingrowing bone marrow cells and may be used with techniques like bone marrow aspirate concentrates (BMACs) [41]. Those matrices are easy to handle and to use trans‐arthroscopically for all types of lesion locations in the knee [64] In a comparative study with HA membrane (Hyalofast ®; Anika Therapeutics) and MFX versus MFX alone, the matrix‐augmented patients demonstrated significant short‐term improvements in pain, stiffness and function when compared to patients treated with MFXs alone [83]. These types of matrices are also useful for osteochondral defect repairs [15, 83]. Furthermore, crosslinked hydrogels have been used for 3D bioprinting and can be loaded with bioactive agents and chondrogeneic cells [23, 41, 44].

*Osteochondral matrix plugs*: Synthetic resorbable cylindrical plugs are nothing new today, but the used materials could be innovative. In common is a slow bony healing into these implants, which could be seen over several years. Slow bony healing might be of importance as many patients respond very well early on with drastic pain relief, even though a large part of the bony area is soft and very little osteogenic healing is seen. A systematic review published in 2015 looked at the use of a synthetic implant made from a polylactide‐coglycolide copolymer (Trufit®; Smith and Nephew) and reported clinical improvement at 12‐month follow‐up [106]. However, in the longer follow‐up, a deterioration of the early improvement was shown [106]. The scaffold was withdrawn from the market in 2013 due to those negative results.

Recently, results have also been presented by the use of a rigid biphasic, biodegradable implant composed of calcium carbonate in aragonite crystalline form [24] as part of the bone phase while the cartilage phase is a composite of modified aragonite and HA (Agili‐C®; Cartiheal) [24]. Experimentally, chondrocytes have been shown to migrate into this scaffold producing extracellular matrix (ECM) rich in collagen type II and aggrecan and lacking collagen type I. Furthermore, the formation of a layer of progenitor‐like cells on the surface of the implant has also been seen [24]. In 2023, a randomized study was published where 251 patients had been randomized to either the aragonite‐based implant or debridement/MFX control arm in a 2:1 ratio [3]. Evaluation was performed at 6, 12, 18 and 24 months, and the implant group showed a statistically superior outcome in the primary endpoint and all secondary endpoints at each follow‐up [3]. At 24 months, 88.5% of the implanted group had a minimum of 75% defect fill on magnetic resonance imaging (MRI) as compared with 30.9% of the controls. The failure rate was 7.2% for the implant group versus 21.4% for control [3].

Another implant that is also addressing both the cartilage and bone area is the bi‐ and triphasic implant Maioregen® (Fin‐Ceramica) [60] that from the start consisted of 6 mm thickness but now exists in both 4 and 2 mm thickness, useful for different degrees of osteochondral depth [60]. In a multicentre randomized study, 100 patients with chondral and osteochondral lesions were treated and evaluated for up to 2 years and randomized to either the biomimetic scaffold or BMS. No statistically significant differences were found compared to BMS alone for chondral lesions, but statistically significant better results were found for deep osteochondral lesions, as well as for sport‐active patients [60].

*Thermogels*: Thermogels are injectable and can be used to fill cartilage defects and then be stabilized ¨in situ¨ [17]. Fundamental for a tissue repair after injury is the formation of a blood clot functioning as the natural scaffold for cell ingrowth. BMS techniques rely on blood clot formation for ingrowth of chondrogeneic cells. Subsequently, an improvement of blood clot formation could be of importance. One method is based on the use of so‐called thermogels [17]. The natural blood clot that is formed has a tendency to shrivel, which means that the clot does not fill the area of injury all the way to the edges and healing can thus be insufficient. By stabilizing the blood clot so that it retains its volume and makes contact with the surrounding cartilage surfaces, the cells from the bone marrow are then able to produce a more complete healing tissue. One way to do this is to add a soluble polymer matrix consisting of the polysaccharide chitosan to the not yet clotted blood [17]. This results in clot formation with good volume and strength. In a randomized study, the augmented blood technique (CARGEL Bioscaffold® (CB) formerly BST‐CarGel®; Smith & Nephew, United Kingdom) resulted in greater lesion filling and superior repair tissue quality compared with MFX treatment alone [98]. Clinical benefit was equivalent between the groups at 12 months. However, at 5 years, the CARGEL bioscaffold® treatment resulted in sustained and significantly superior repair tissue quantity and quality over MFX alone [93].

*Chondrogeneic tissue repairs*: In the 1990s, chondrogeneic tissue repairs were popular, such as perichondral [8, 53] and periosteal [2, 78] resurfacings. With those tissues, especially with perichondrium, there were problems with ossification and due to such facts loosening of grafts [9]. Those effects could be a normal result of endochondral ossification and difficult to control in in situ situations. The best results were seen in young patients [75]. There is only little clinical use today and those techniques have more or less disappeared from the treatment market. Periosteum may still be important in young patients with high regenerative abilities in acute trauma situations while the cell implants may have more importance in the older patients [75]. However, even though the cambium layer becomes thinner with increasing age, the remaining periosteal‐derived cells from old people were after several passages superior in producing bone or cartilage compared to bone marrow MSCs from a similar source [63].

*Autologous and allogeneic osteochondral grafts*: Autologous osteochondral grafts (mosaicplasties) were popular in the 1990s. They are still used but have lost little in popularity last years. However, when used, the implants show long‐term durability compared to MFXs [96]. Instead, the use of osteochondral allografts has considerably increased during recent years. A problem is still lack of donors and risk for disease transmission, but there is a high percentage of success. In a recent study, osteochondral allografts demonstrated significant improvements in clinical outcome scores and good durability with successful outcomes in 75% of the patients at 12.3 years after surgery [4]. Patellofemoral lesions are associated with decreased clinical improvement and more frequent reoperations [4].

To this category of implants belongs also the off‐the‐shelf products composed of donated human decellularized hyaline cartilage and cancellous bone. Those implants are in pre‐cut sizes to accommodate lesions of varying sizes and shapes and being allografts not associated with any donor site morbidity [33]. However, difficulties have been seen with the use of such implants. In one study, a 72% failure rate within the first 2 years of implantation was seen [33] and in another implant, survivorship was 61% at 2 years [54]. Female gender was independently predictive of failure, with a hazard ratio of 9.4 [54].

### Chondrogeneic cell implantations

#### BMAC

BMAC is another source of MSCs [41]. Those cells have been shown to interact within HA‐based scaffolds in such a manner that promotes cellular adhesion, proliferation, migration and the generation of ECM components. However, patient age may change the quantity and quality of BMAC obtained and in a study it was shown that also harvest site and age can affect the quality of BMAC [20]. MSCs obtained from iliac crest and proximal tibia present comparable mesenchymal markers expression as well as osteogenic and chondrogeneic differentiation potential, but iliac crest BMAC presents a four times higher number of mononucleated cells with significantly higher clonogenic capacity compared to the tibia [20]. BMAC was also shown to have a three times higher number of mononucleated cells in younger patients [20].

#### Chondrocyte implantations

**Figure 2:**
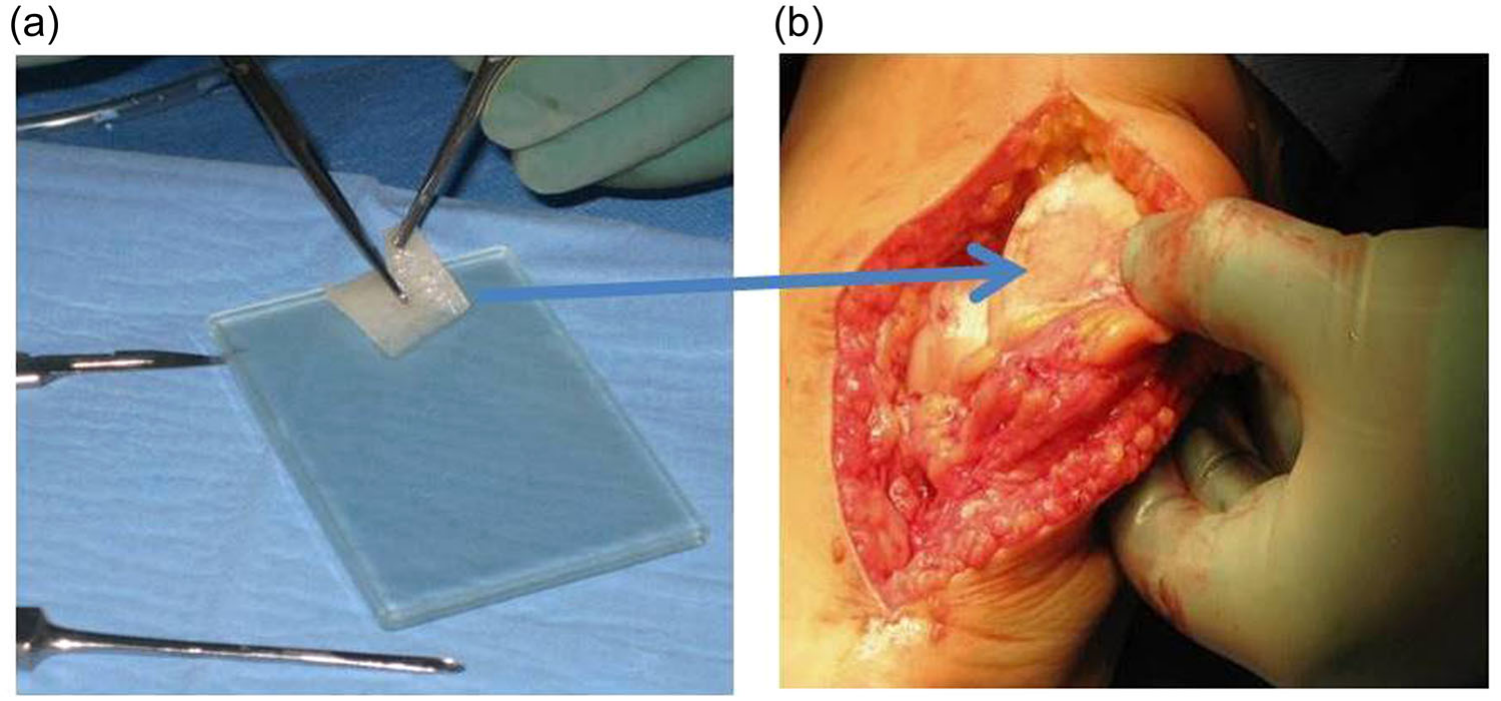
(a) A cell‐seeded hyaluronic acid scaffold seeded with autologous chondrocytes is sized to fit into a debrided patella cartilage defect. (b) The cell‐seeded hyaluronic acid scaffold has been implanted into the patella defect and covered by a layer of fibrin glue.

The first autologous chondrocyte implantation (ACI) was performed in Gothenburg, Sweden, in October 1987 [12]. Since then, the technique has changed from a first‐generation ACI until now fifth‐generation ACI. The first [12] and second [42] generations of ACI used a cell suspension injected under a cover of either periosteum or collagen membrane. The third‐generation ACI is either cells in vitro cultured on a membrane [13] or grown on a porous scaffold [67] (Figure 2a,b). With the addition of different matrices, of which several have been of animal origin, the use of those implants has been difficult in some countries [43]. Products of biological origin have a definite restriction for various religions [43].

The fourth‐generation ACIs involve one‐stage chondrocyte implantations [65], while the fifth‐generation ACI will be 3‐D‐printed cell alternatives.

Mostly used today are different third‐generation ACI products, but as it is difficult to get approval for a larger use of those technologies, there is now a tendency for more use of the new fourth‐generation ACIs. One such fourth‐generation ACI is the use of cartilage fragments as a source for migrating chondrocytes [65]. It was first presented as CAIS® (cartilage autograft implantation system; DePuy/Mitek) with harvest of autologous cartilage fragments with a special instrument that distributed the harvested fragments on a resorbable membrane to be implanted into the cartilage defects [65]. CAIS® first demonstrated proof of concept in animal studies [37] and was followed by two randomized studies [25, 97], showing superiority of implanted fragments versus microfracturing at 2 years follow‐up. Now there are different variants of the use of fragmented/minced cartilage like the Autocart®‐product [40] (Arthrex and techniques like CAFRIMA (cartilage fragment implantation membrane augmented) [11]. Also, use of allogeneic fragment cartilages is available from young donors [34]. Significantly better repair is seen when mixing allogenic young cartilage fragments with fragments from old donors, compared to only the use of old cartilage fragments when treating cartilage defects as seen in a rabbit model [7].

With the minced cartilage, the main purpose is to get the cells out of their domains to repopulate new areas bridging defective cartilage areas with new matrix. Chondrocytes or chondroprogenitors migrate to the site of injury and repair the injury by synthesizing the lost ECM. To migrate, the cells need to remove the surrounding ECM by expressing proteolytic enzymes. Addition of enzymes like trypsin to the surrounding lesion walls enhances migration out from surrounding cartilage to support the cell's migration from implanted fragments [92].

The scaffold surrounding the implanted fragments may differ, but as the use of such fragments is not a cell manipulation, the use has increased fast in the last few years. Other fourth‐generation ACI variants, include when you make a direct isolation of chondrocytes in the operating theatre and mix those cells with autologous iliac crest bone marrow aspirates as has been done in the INSTRUCT study [94]. A similar one‐stage ACI is when one isolates chondrocytes including their pericellular matrix, the so‐called chondrons and mixes them with allogeneic MSCs as in the IMPACT study [30]. Another way to avoid two‐stage procedures for chondrocyte implantation is to use allogeneic chondrocyte therapies [49]. It is possible to obtain viable chondrocytes from cartilage harvested from cadaveric donors to obtain similar cell numbers and viability compared to cells of living donors [79].

### Unloading of cartilage repair

Mechanical unloading approaches are suggested to be beneficial in preserving the chondrocyte phenotype [111]. In OA, catabolic processes degrade the cartilage matrix, and the composition and viscoelastic properties of the matrix produced by chondrocytes will then be altered [111]. Pathological loading of the cells and their matrix will then be created by these load changes. Chondrocytes are influenced by their mechanosensitive receptors and channels that activate a complex network of downstream signalling pathways that may develop into an OA [112]. Unloading the diseased joint is subsequently important both at a molecular level and due to malalignment with pathological joint biomechanics [46]. Most used are unloading osteotomies [29], but there is also an increased interest in the use of joint distractions [51]. Joint distraction is a temporary mechanical separation of the bones at a joint with external fixators. In clinical studies on the knee, significant clinical and structural improvements over 2 years have been reported [110]. Both cartilage volume increases, as well as thinning of the subchondral cortical bone plate, and decrease of subchondral trabecular bone density were noted after 2 years follow‐up [69]. Furthermore, in a recent report, the structural changes remained improved at 10 years follow‐up [52].

Finally, there will always be nonresponders to local cartilage repair methods. For such nonresponders, local minimetal and synthetic implants are available [47, 70].

### Future steps

With the interest to use induced pluripotential stem cells, the possibility to 3D print cells in varied types of bio‐inks in different layers is now studied [77]. With 3D MRI evaluations, a precise estimation of lesion site may be done. With the use of a bio pen, the exact number of cells in different layers may then be printed into the lesion area by arthroscopy [80].

When talking about cartilage repair, we have been focused on the use of true committed chondrocytes and chondrogeneic stem cells of varied origin. However, it is well known that macrophages can play a significant role in modulating joint inflammation, and thus severity of cartilage destruction, via various secreted mediators [35]. Macrophages are immune cells found in synovial lining, with different roles depending on their subtypes. Those cells may turn into either proinflammatory (M1) or anti‐inflammatory (M2) phenotypes. The M2 cells are associated with tissue lesion healing by the production of different cytokines [35]. Of extra interest and importance is that under the stimulation of certain biomaterials, M2 macrophages could be activated, release cytokines and exert an immunomodulatory effect on tissue healing and osteogenic differentiation in vitro [28]. Subsequently, we need to know even more about supporting scaffolds that we are in use to have the best scaffold chondrogeneic stimulation and stability.

## SUMMARY AND CONCLUSION

The nonoperative options available to use when treating cartilage lesions are mainly based on anti‐inflammatory and local growth‐related effects. PRP, HA and stem cell injections have unpredictable results and mainly temporary effects and are more suitable for OA treatments than local repairs.

To treat operatively cartilage lesions in 2024, still simple BMS techniques are used. However, such techniques are indicated only for small lesions and then the BMS should preferably be performed via subchondral thin microdrillings instead of MFXs. With slightly larger lesions, augmentation of the bone marrow‐stimulated area with a supporting scaffold is an option often used, as it is easy to manage and not too expensive. For larger lesions, cell‐seeded alternatives such as ACI third generations are still popular. The fourth‐generation ACIs with minced autologous and allogeneic cartilage are gaining popularity, as they are fast and easily used techniques with reasonable pricing. Young donor allogeneic cell sources for chondrocytes and mesenchymal cells with large‐scale productions ensuring a stable chondrogeneic quality will probably become the future option [32]. Allogeneic sources for PRP and stem cell lines could also be future alternatives when adding growth factors to the local repair [16].

## AUTHOR CONTRIBUTIONS

I am the only author, and I have done all research and writing all by myself.

## CONFLICT OF INTEREST STATEMENT

The author is a member of the advisory board of: Episurf Medical AB, Xintela AB, Magellan Stem Cells Pty Ltd., Cline Scientific, Askel Healthcare Ltd. and Vanarix SA. Participation speaker's bureau for Arthrex and Anika Therapeutics. Share holder: Abliva AB and Cline Scientific AB. Member editorial board: *Osteoarthritis & Cartilage*. Editor‐in‐Chief: *CARTILAGE*.

## ETHICS STATEMENT

No ethical committee approval or patient consent was needed due to the nature of the study.
